# The soft rock can promote the improvement of aeolian sandy soil in Mu Us Sandy Land, China

**DOI:** 10.1038/s41598-023-38928-7

**Published:** 2023-07-21

**Authors:** Zhen Guo, Juan Li, Yang Zhang, Huanyuan Wang, Wanying Li

**Affiliations:** 1 Technology Innovation Center for Land Engineering and Human Settlements, Shaanxi Land Engineering Construction Group Co., Ltd. and Xi’an Jiaotong University, Xi’an, 710075 China; 2 Shaanxi Provincial Land Engineering Construction Group Co., Ltd., Xi’an, 710075 China; 3 Institute of Land Engineering and Technology, Shaanxi Provincial Land Engineering Construction Group Co., Ltd., Xi’an, 710021 China

**Keywords:** Ecology, Environmental sciences, Solid Earth sciences, Engineering

## Abstract

This study focuses on the significance of improving the land degradation of Mu Us Sandy Land to increase cultivated land area and promote ecological green development. The research objects were four kinds of mixed soils, and rhizosphere soils were collected during the crop harvesting period. The volume ratio of soft rock to sand was 0:1 (control check, CK), 1:5 (composite soil one, PS1), 1:2 (composite soil two, PS2), and 1:1 (composite soil three, PS3). The results showed that the large aggregates were primarily mechanically stable aggregates, while the small aggregates were mainly water-stable aggregates. The soft rock promoted the increase of clay and silt content in sandy soil, and the soil texture changed from sand to loam. The contents of organic matter, available phosphorus, and available potassium increased significantly under PS2 and PS3 treatments, but there was no significant difference between them. Total nitrogen had no significant difference among treatments. *Actinobaciota*, *Proteobateria,* and *Chloroflexi* were the dominant bacteria in rhizosphere soil, accounting for about 75% of all microorganisms. At the Genus level, the soft rock contributes to richer species composition. The diversity index, evenness index, and richness index was higher in PS1, and the available phosphorus and available potassium content promoted the increase of diversity. Therefore, when the proportion of soft rock and sand compound soil is between 1: 5 and 1: 2, it can be used as an important basis and technical parameter for Mu Us Sandy Land improvement.

## Introduction

The process of land desertification has emerged as a critical ecological and social issue with significant implications for the survival and progress of humanity^1^. The desertification area in the world has reached more than 400 million hm^2^, of which the seriously desertified land is nearly 20 million hm^2^, and is still expanding at the rate of 300,000 hm^2^ per year^2,3^. China faces a scarcity of land resources, with a per capita cultivated land area that is only a quarter of the world average^4^. In consideration of land resources and economic development, China has established a goal to safeguard cultivated land by implementing measures such as controlling the total amount of cultivated land, enhancing the quality of cultivated land, and guaranteeing food security. Accelerating the rehabilitation of desertified land is crucial for both promoting economic development in desertification areas and fostering harmonious coexistence between humans and nature^5^, as well as maintaining a stable society.

Throughout human history, efforts have been made to prevent and control desertification through both theoretical and practical means. Presently, the management of desertified land involves implementing measures such as vegetation, engineering, and chemicals^6,7^. The theory of soil organic rebuilding, which is crucial in combating desertification, has proven to be effective in land restoration projects^8^. The soil organic reconstruction is a technical system that involves converting non-agricultural land into farmland, upgrading low-level land, and implementing informationalization land projects through adjustments, reorganization, and replacement. The ultimate goal is to create a physical space that supports the survival and multiplication of organic organisms, known as “Pure Land”. The specific project was to combine the local soft clay minerals into the sand to create a well permeable composite soil, and to establish water saving irrigation methods, thus increasing the productive capacity of the land and promoting the sustainable utilization of sand^9^. Soft rock is a type of loose rock that belongs to the clastic rock series of continental facies. It has a low degree of diagenesis, poor sand grain cementation, low structural strength, and is susceptible to soil erosion^10^. The main components of the soft rock are quartz, calcium montmorillonite, potash feldspar and calcite, and other lower contents^11^. The soft rock is concentrated in the Ordos Plateau in the northern part of the Loess Plateau, bordering Shanxi, Shaanxi, and Inner Mongolia. According to the degree of soil cover, it can be divided into three types of areas, namely, the exposed soft rock area, the soil cover area, and the sand cover area^12^, with a total area of 16,700 km^2^. Both soft rock and sand were exposed to erosion and after exposure, wind erosion was reduced while soil erosion was improved^13,14^. The study of Guo et al.^15^ on the mechanics of soft rock indicates that when the soft rock was added to the sand, the fine grains took up the place of the big grains in the sand, which resulted in the increase of the distance of the grains, which will result in the tension stress. Li et al.^16^ research on sand soil indicated that the addition of soft rock could increase the weight ratio and the structural stability of sand. The results of Wang et al.^17^ research on the hydraulic properties of soft rock and sand indicated that when the ratio of soft rock was higher, there was no significant change in wilting coefficient, and the ability to retain water in the field was improved.

Previous research on composite soil composed of soft rock and sand has primarily focused on aggregate structure, mechanical properties, and soil moisture^13–17^. However, there is a lack of collaborative research on the physical, chemical, and biological properties of this type of soil. In particular, the biological research on soft rock and composite sand soil was insufficient. Soil physical and chemical properties are essential factors that determine soil fertility and quality. These properties serve as the foundation for crop growth and development, making them crucial in agricultural production^18^. The variation in the proportion of soft rock will lead to a greater range of options when attempting to restore productivity. For this reason, the desert area in Yulin of Mu Us Sandy Land is studied, and different mixing ratio of soft rock is designed to form mixed soil. The main objectives of this study are to (1) reveal the physical and chemical characteristics of rhizosphere soils of different compounded soils; (2) demonstrate the biological characteristics of rhizosphere soils of compounded soils; and (3) clarify the agglomeration characteristics of compounded soils.

## Results

### Water stable aggregate and mechanical stable aggregate content of composite soil

Tables 1 and 2 display the percentage of soil aggregates larger than 0.25 mm obtained through the methods of overdry screening and wet screening, respectively. The majority of the macroaggregate content was found to be in the size range of greater than 5 mm when using the dry screening method. The PS3 treatment showed a significant increase compared to other treatments, with a percentage increase of 18.11, 25.2, and 20.64 compared to CK, PS1, and PS2, respectively. The content of small aggregates was opposite to that of large aggregates (Table 1). The content of aggregates larger than 0.5 mm was found to be smaller when treated with wet sieve compared to dry sieve. The aggregates treated with wet sieve were mainly distributed in the particle size range of < 0.25 mm, accounting for more than 70% of the total. However, there was no significant difference observed among all the treatments as shown in Table 2. The treatment PS2 resulted in the lowest water-stable aggregates larger than 5 mm, while treatment PS1 resulted in the highest water-stable aggregates smaller than 0.25 mm.

**Table 1:** Mechanical stability aggregate content of composite soil (%).

Treatments	> 5 mm	2–5 mm	1–2 mm	0.5–1 mm	0.25–0.5 mm	< 0.25 mm
CK	29.17 b	7.83 b	7.43 a	7.80 a	16.67 a	31.09 a
PS1	22.08 b	8.77 ab	6.48 a	8.71 a	14.42 a	39.54 a
PS2	26.64 b	10.78 a	8.91 a	8.35 a	14.58 a	30.74 a
PS3	47.28 a	12.20 a	8.43 a	7.83 a	8.68 b	15.58 b

CK, PS1, PS2, and PS3 represent the volume ratio of soft rock to sand as 0:1, 1:5, 1:2, and 1:1, respectively. The sample quantity is 12. Different letters on the column indicate significant differences among different treatments (*P* < 0.05).

**Table 2:** Water stability aggregate content of composite soil (%).

Treatments	> 5 mm	2–5 mm	1–2 mm	0.5–1 mm	0.25–0.5 mm	< 0.25 mm
CK	2.46 a	0.96 a	2.16 ab	5.68 a	16.94 a	71.80 a
PS1	2.26 a	1.80 a	1.24 b	1.76 b	15.24 a	77.70 a
PS2	1.10 b	1.44 a	3.20 a	5.04 a	15.06 a	74.16 a
PS3	1.80 ab	1.96 a	3.62 a	4.42 a	13.56 a	74.64 a

CK, PS1, PS2, and PS3 represent the volume ratio of soft rock to sand as 0:1, 1:5, 1:2, and 1:1, respectively. The sample quantity is 12. Different letters on the column indicate significant differences among different treatments (*P* < 0.05).

### Microstructure of microaggregate in rhizosphere soil

**Figure 1:**
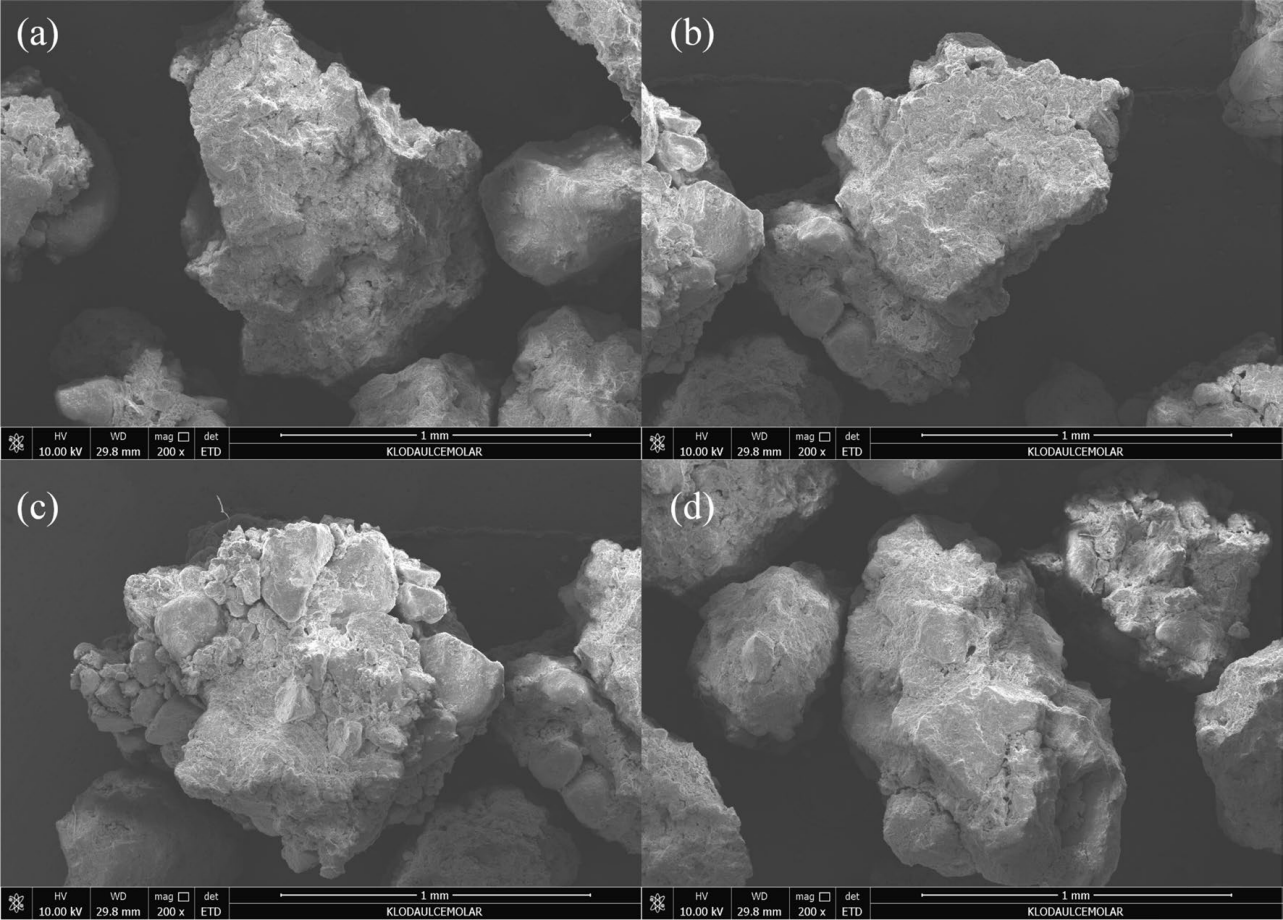
Microstructure of aggregates of different composite soils with particle size < 0.25 mm. (**a**)–(**d**) represent the volume ratio of soft rock to sand as 0:1, 1:5, 1:2, and 1:1, respectively.

The aeolian sandy soil is characterized by a loose soil structure with single granules and a lack of cohesion (Fig. 1a). As the proportion of soft rock increased, the microaggregate structure of composite soil with a grain size of less than 0.25 mm became more apparent. A few sand particles adhered to other sand particles, resulting in a reduction in the distance between soil particles within this grain size range (Fig. 1b). Upon observation, it is evident that the 1:2 composite soils have clay granules adsorbed onto sand grains in a wrapping state. Furthermore, the large pores are filled with small particles (Fig. 1c). As the proportion of soft rock increased, the clay particles gradually filled the macroporosity between the sand grains, resulting in a more compact aggregate structure with clear margins (Fig. 1d). It can be seen that the soft rock promotes the formation and development of aeolian sand agglomeration structure.

### Soil texture of rhizosphere soil

**Figure 2:**
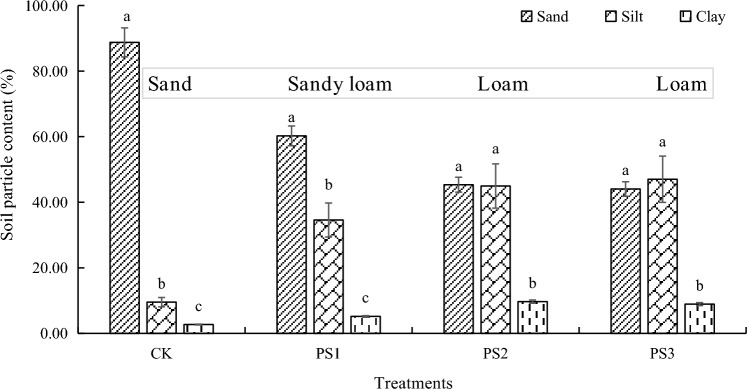
Soil texture of different compound soils. CK, PS1, PS2, and PS3 represent the volume ratio of soft rock to sand as 0:1, 1:5, 1:2, and 1:1, respectively. Different lowercase letters indicate significant differences in soil particles between the same treatments (*P* < 0.05).

Compared to CK treatment, the addition of soft rock resulted in a significant increase in soil clay and silt content, while sand content was significantly decreased. The soil texture was changed from sandy to loam (Fig. 2). In the study of PS2 and PS3, as the proportion of soft rock increased, there was no significant change in soil texture or particle composition.

### Nutrient content of rhizosphere soil

**Figure 3:**
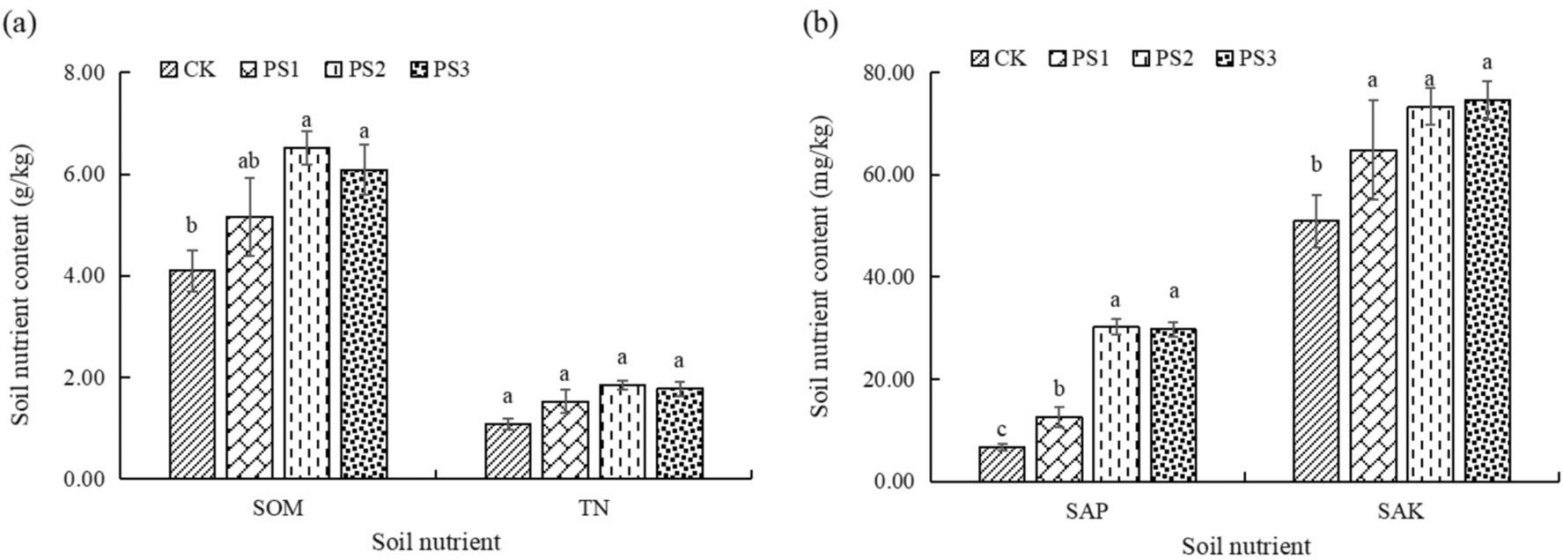
Soil nutrient content of different compound soils. CK, PS1, PS2, and PS3 represent the volume ratio of soft rock to sand as 0:1, 1:5, 1:2, and 1:1, respectively. SOM, TN, SAP, and SAK represent soil organic matter, total nitrogen, soil available phosphorus, and soil available potassium, respectively. Different lowercase letters indicate significant differences between treatments (*P* < 0.05).

The amount of organic matter in PS2 and PS3 was higher than that of CK but not in PS1 (Fig. 3). There was no significant difference in total nitrogen content among the treatments. The proportion of soft rock has a positive correlation with the content of available phosphorus and available potassium. The nutrient content increases significantly with 1:2 and 1:1 treatments. The study found no significant difference in soil organic matter and total nitrogen between CK and PS1 treatments. However, the PS1 treatment showed a significant increase in available phosphorus and available potassium compared to CK treatment.

### Composition of soil bacterial community

**Figure 4:**
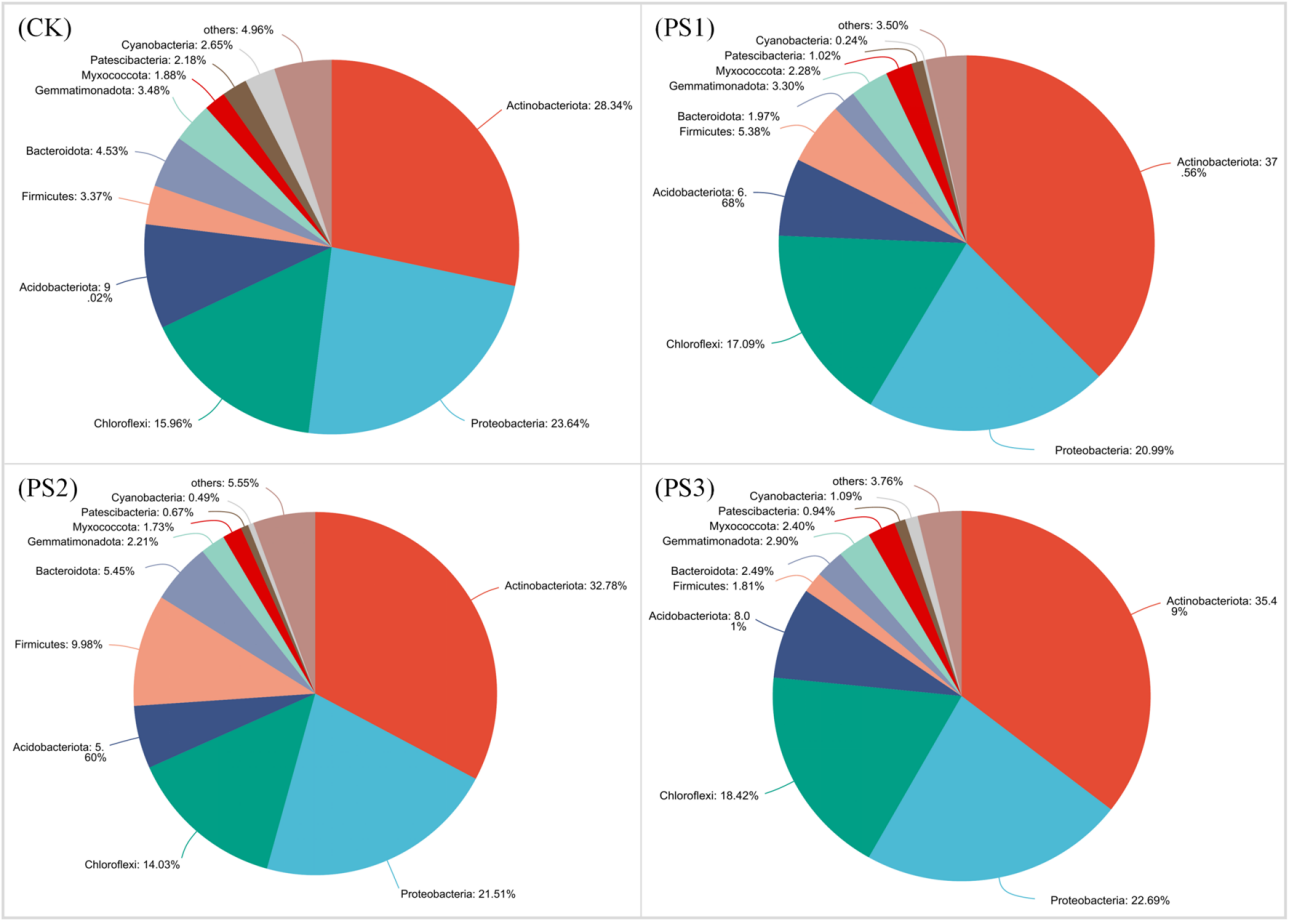
Bacterial community composition based on Phylum level. CK, PS1, PS2, and PS3 represent the volume ratio of soft rock to sand as 0:1, 1:5, 1:2, and 1:1, respectively.

At the Phylum taxonomic level, the *Actinobacteriota*, *Proteobacteria*, and *Chloroflexi* were found to be the dominant phyla in each treatment, making up 67.94% to 76.60% of the total bacteria (Fig. 4). Compared with CK, the abundance of *Actinobacteriota* in PS1, PS2, and PS3 treatment has increased by 9.22, 4.44 and 7.15 percentage points, respectively. As the proportion of soft rock increases, the abundance of *Actinobacteriota* initially increases, then decreases, and finally rises again. The abundance of *Proteobacteria* showed a decreasing trend after adding soft rock, but the decreasing amplitude decreases with the increase of soft rock proportion. Compared with CK, the abundance of PS1, PS2, and PS3 decreased by 2.65, 2.13, and 0.95 percentage points, respectively. The *Chloroflexi* had a similar trend with *Actinobacteriota*.

**Figure 5:**
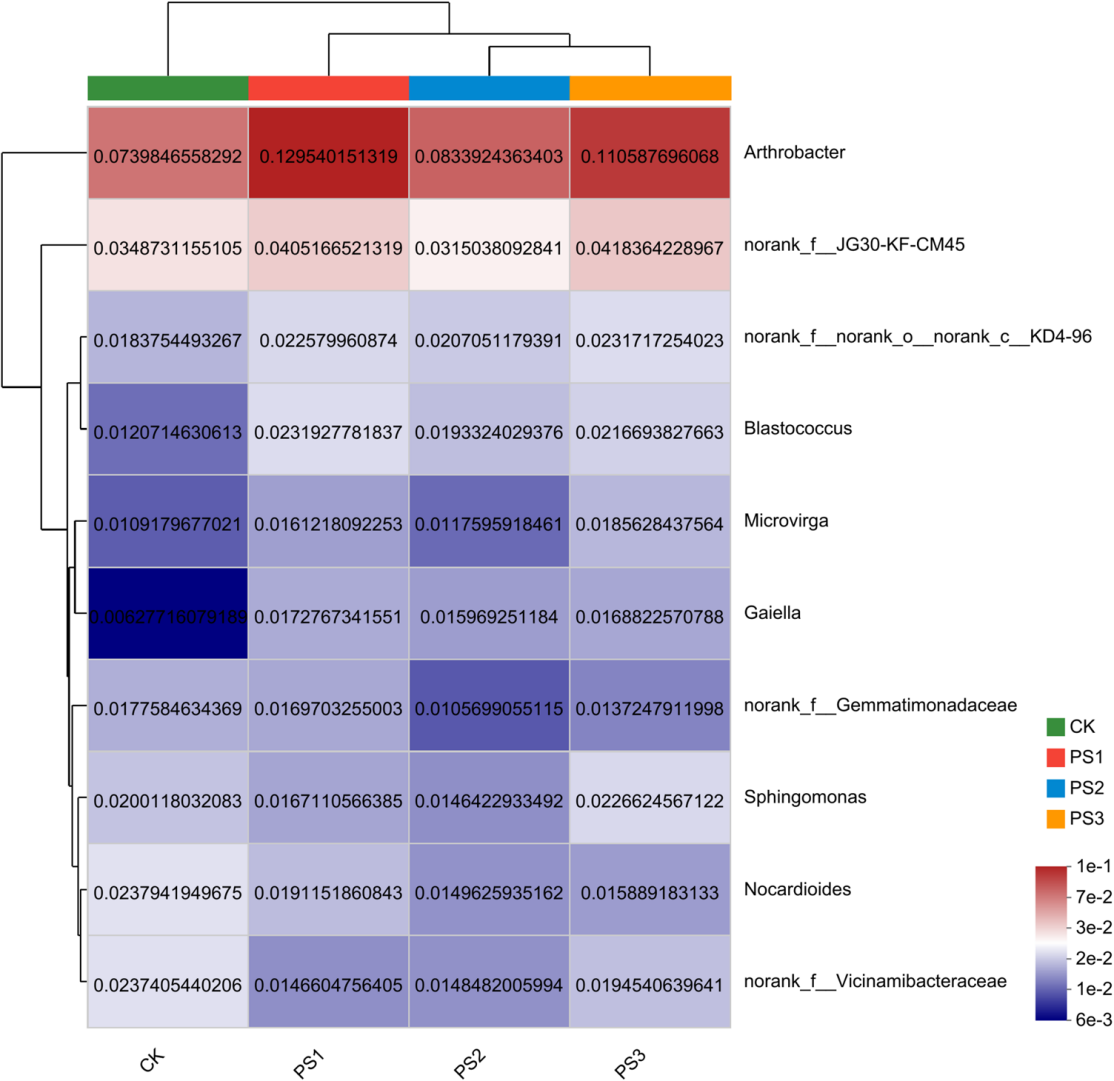
Bacterial community composition based on Genus level. CK, PS1, PS2, and PS3 represent the volume ratio of soft rock to sand as 0:1, 1:5, 1:2, and 1:1, respectively.

At the Genus level, the dominant bacteria were consistent across different treatments, however, a greater variety of unique genera were observed. The dominant bacteria in CK treatment were *Arthrobacter*, *norank_f__JG30-KF-CM45,* and *norank_f__norank_o__norank_c__KD4-96*. When compared to CK treatment, the dominant genera in PS1, PS2, and PS3 treatment exhibited a fluctuation trend of initially increasing, followed by decreasing, and then increasing again with the increase in soft rock (Fig. 5). In addition, the correlation heatmap also showed that the bacterial community structure treated by PS2 and PS3 was similar, and the two groups clustered into one class. Moreover, the three dominant genera was different from each other and clustered into one category.

### Soil bacterial diversity

The study found that the bacterial coverage rate of soil samples was between 94.70% and 97.96%. This suggests that the sequencing data used in the study is a reliable reflection of the species and fundamental structure of the sampled flora. There was no significant difference between the Shannon index and Shannoneven index after compounding soft rock and sand (Table 3). The Simpson index, Ace index, and Chao index had the same trend, but PS1 was significantly higher than CK. Compared with CK treatment, the Simpson index, Ace index, and Chao index increased by 0.53%, 13.32%, and 13.52%, respectively. Simpson even index increased by 150%, 79.92%, and 84.82% compared with CK, PS2, and PS3, respectively.

**Table 3:** Bacterial diversity index in different mixed soils.

Treatments	Diversity index		Richness index		Evenness index		Coverage (%)
	Shannon	Simpson	Ace	Chao	Shannon even	Simpson even	
CK	6.17 a	0.0122 b	3710 b	3713 b	0.7671 a	0.0190 b	97.56
PS1	6.33 a	0.0175 a	4204 a	4215 a	0.7984 a	0.0475 a	97.49
PS2	6.20 a	0.0172 a	3966 ab	3988 ab	0.7764 a	0.0264 b	97.96
PS3	6.24 a	0.0156 ab	4120 ab	4151 ab	0.7796 a	0.0257 b	97.40

CK, PS1, PS2, and PS3 represent the volume ratio of soft rock to sand as 0:1, 1:5, 1:2, and 1:1, respectively. Different lowercase letters indicate significant differences between treatments (*P* < 0.05).

### Relationship of soil nutrients in the rhizosphere and microbial diversity

According to the results of Pearson correlation analysis presented in Table 4, there was a significant positive correlation between the Simpson index and the SAP and SAK content. This suggests that an increase in SAP and SAK content can enhance soil microbial diversity. The results of the study showed a positive correlation between the Simpson index and clay and silt particles, while a negative correlation was observed with sand grains. The Simpson even index showed a negative correlation with the SAP, SAK, clay, and silt contents. The study suggests that incorporating soft rock can enhance microbial diversity in sandy soil and reduce its uniformity.

**Table 4:** Correlation analysis between soil nutrients and microbial diversity.

Item	SOM	TN	SAP	SAK	Clay	Silt	Sand
Shannon	−0.2083	−0.0354	−0.3259	−0.3211	−0.2627	−0.2632	0.2759
Simpson	0.4508	0.2154	0.5920*	0.6558*	0.5027*	0.5087*	−0.5124*
Ace	−0.0316	−0.0026	−0.0208	0.0760	0.0311	0.0781	−0.0727
Chao	−0.0173	0.0138	−0.0176	0.0826	0.0467	0.0913	−0.0846
Shannoneven	−0.3057	−0.0701	−0.4935	−0.5558**	−0.4106	−0.4432	0.4568
Simpsoneven	−0.4065	−0.1176	−0.5998*	−0.7256*	−0.6080*	−0.6599**	0.6733**

SOM stands for soil organic matter, TN stands for total soil nitrogen, SAP stands for soil available phosphorus, and SAK stands for soil available potassium.

*Significant *P* < 0.05.

**Significant *P* < 0.05.

## Discussion

### Influence of soft rock on physical properties of sandy soil

Soil agglomeration plays a crucial role in soil composition, as it facilitates the coordination of water, fertilizer, air, and heat within the soil. It also impacts the types and activities of soil enzymes, helps to maintain and stabilize the loose maturation layer of the soil, and ultimately has a direct effect on plant productivity^19^. Research has demonstrated that the stability of soil structure can be impacted by tillage methods and soil composition due to their influence on the transformation and redistribution between microaggregates and macroaggregates^20,21^. The results showed that the soil structure of the treated soft rock was better than that of CK. The mechanical stable aggregate was mainly more than 0.25 mm, accounting for 60.46–84.42%, and the water-stable aggregate was less than 0.25 mm, accounting for 71.80–77.70%. It showed that water play an important role in developing composite soil. This study collected and analyzed samples at the end of crop harvesting. The soil moisture content was found to be moderate, and the distribution of soil particles was relatively uniform, with a predominance of large aggregates. The increase in plant root exudates and microbial metabolites in the soil promotes the organic glue coupling between soil particles, which enhances agglomeration and transforms particle sizes of less than 0.25 mm into larger agglomerates. This process lays the foundation for further agglomeration^22^. The results were consistent with the research on improving soil structure by planting crops on degraded and desertified land^23^. The results also showed that the microstructure with a diameter of less than 0.25 mm was rich in cementing materials (Fig. 1), which can provide the material basis for the formation of large aggregates. Numerous scholars have acknowledged the significant contribution of clay minerals towards enhancing sandy land. These minerals aid in the accumulation of soil particles, retention of water and fertilizers, and ultimately lead to improved crop yields^12–17^.

### Effects of soft rock on fertility characteristics of sandy soil

Soil fertility is a crucial component of cultivated land and plays a significant role in determining soil quality and the sustainable use of cultivated land resources^24^. The nutrient content of the mixed soils of 1:5, 1:2, and 1:1 showed an overall upward trend, which was due to the implantation of inorganic colloids (soft rock) in aeolian sandy soil, the increase in the content of soil silt and clay (Fig. 2), and the easy combination with soil nutrients to form organic–inorganic complexes, which provides physical protection for nutrients. On the other hand, tillage management and organic matter input, as well as the decomposition of potato root systems, have been shown to improve the overall soil bio-habitat conditions^25^. The effect of 1:2 compound soil was better, and the texture was loam, suitable for crop planting. The results indicate that the optimal ratio of soft rock can significantly alter the availability of nutrients and substantially impact soil properties. According to relevant studies, the 1:2 composite soil has a uniform particle composition, moderate distribution, and meets the suitable conditions for crop growth. Additionally, it has better aeration and permeability^26,27^.

### Effects of soft rock on biocharacteristics of sandy soil

Changes in the abundance of predominant bacterial phyla were observed in sandy soil after the introduction of soft rock, while the overall composition of the bacterial community remained unchanged at the phylum level. This was the same as that of the research on the effect of plantation restoration on the microbes in Mu Us Sandy Land^28^. *Actinobacteria* was the dominant bacteria in this study and had the highest abundance in different treatments. Research has demonstrated that *Actinobacteria* is a prevalent soil parasite with robust adhesion capabilities. It has the potential to serve as a storage bacteria while its mucus secretion can effectively bind sand particles. Additionally, its filamentous body structure promotes soil structure stability^29^. The results showed that soft rock had a certain effect on the microbial community structure and promoted the formation of agglomeration structure. Diversity analysis also showed that the appropriate addition of the soft rock could effectively promote the increase of bacterial diversity, abundance, and uniformity. The available phosphorus and available potassium were positively correlated with the diversity index. According to research, soft rock contains primary minerals such as calcium, magnesium, potassium, sodium, and other common elements. These minerals are released through weathering, providing nutrients that can be absorbed by plants and microbes^30^. Therefore, the increase in nutrient content was promoted by the increase in microbial diversity and abundance.

## Conclusions

Soil properties are affected by land use patterns, vegetation, and meteorological factors. After years of cultivation, the characteristics of different proportions of compounded soils have been increasingly improved. Aeolian sand has no cohesion between single grains, and its surface is smooth. After adding soft rock, the surface cementation of microaggregate soil particles was strengthened, and the organic attachment was increased. Under normal tillage conditions, soil particles exist in the form of large aggregates. With the increase of water and fertilizer content, small particles can also promote the formation of large aggregates. The soil texture changed from sandy to loam soil, which was more favorable for crop planting. The structure and performance of 1:2 composite soil was more prominent. On this basis, the contents of organic matter, total nitrogen, available phosphorus, and available potassium were rich when the ratio of soft rock and sand was 1:2 and 1:1, but there was no significant difference between them. At different classification levels, *Actinobacteriota* showed absolute superiority. The diversity and richness of microorganisms was improved in the mixed soil, and the mixed soil with 1:5 was better. The contents of available phosphorus and available potassium were positively correlated with microbial diversity and negatively correlated with evenness. Therefore, when the ratio of soft rock to sand was between 1:5 and 1:2, the comprehensive properties of soil were better, which can be used as a better choice for sandy land improvement.

Through this study, we can supplement the biological basis of Mu Us sandy land improvement research and the application scope of soft rock materials. In the use of soft rock to improve sandy land, this paper also provided a wealth of basic theoretical data for scholars to provide theoretical references. In conclusion, applying soft rock to improve aeolian sand soil provides feasible measures to control Mu Us Sandy Land and can be popularized in similar areas. The research results can not only increase the area of agricultural land in sandy areas but also promote the sustainable development of the local agricultural economy and the improvement of the ecological environment.

## Materials and methods

### Study site

The study was conducted in Yulin City of Xiaojihan Township of Shaanxi Province. The research area lies on the southwestern edge of Mu Us Sandy Land. It is situated on the northern side of the North Wind Sand. The study area is located in the temperate continental monsoon climate zone, with annual average temperature of 13 °C, abundant sunshine, annual average sunshine duration of 2390 h, annual frost free period of 165 d, dry climate, perennial drought, and little rain, and annual average precipitation of 300 mm. Due to the strong northwest wind, it is easy for sand storms to appear in the spring. The type of soil in the study area is aeolian sandy land with a loose texture and poor nutrients. The mineral component of the soil is made up of fine grains of sand, which contains less clay and silt. Most of the plants are xerophytes and middle xerophytes.

### Experimental materials

The types of soft rock are white, gray, purple, pink, and other types. Both the soft rock and sand (sandy soil) used in this study was collected from Dajihan village, Yuyang District, Yulin City, Mu Us Sandy Land. The formation process of soft rock is influenced by various factors, including geological structure, climate, and biological action. As a result, soft rock exhibits diverse morphology and properties. The soft rock used in this study was purplish-red. It consisted of a loose rock formation known as an interlayer, which comprised thick sandstones, sand shales, and argillaceous sandstones. These rocks belonged to the Paleozoic Permian (approximately 250 million years ago) as well as the Mesozoic Triassic, Jurassic, and Cretaceous periods. The soft rock referred to in this study is a continental clastic series characterized by low pressure, a low degree of diagenesis, and low structural strength. The minerals in soft rock mainly contain quartz and montmorillonite, while the minerals in sand are mainly quartz. The basic properties are shown in Table 5.

**Table 5:** The basic properties of soft rock and sand.

Material	SOM (g/kg)	TN (g/kg)	SiO_2_ (%)	FeO (%)	CaO (%)	K_2_O (%)O	Al_2_O_3_ (%)
Soft rock	0.78	0.23	64.67	10.12	1.64	3.00	12.83
Sand	3.32	0.14	78.05	2.64	2.08	2.16	11.84

### Test design

In order to simulate the land condition of soft rock and sand mixed layer in Mu Us Sandy land, a field experiment plot was set up in Mu Us Sandy Land in Yulin, China. The field was set up in 2010 and has been planted for 13 years. A mixture of soft rock and sand was placed on the test ground at 0 to 30 cm depth. The soil layer below 30 cm was primitive aeolian sand soil. The soft rock and sand were mixed to form composite soil according to the volume ratio of 0:1, 1:5, 1:2, and 1:1 (soft rock: sand). CK (control check), PS1 (composite soil one), PS2 (composite soil two), and PS3 (composite soil three) represents these proportions in order. Each treatment was set up with 3 replicates and a total of 12 test plots. The experimental fields were planted at the beginning of April, and they were harvested in mid to late September according to a single crop per year with potato and maize rotation. During the cultivation period, only chemical fertilizers and no organic fertilizers were added. The fertilizer types tested in the test field were urea, diammomium phosphate, and potassium chloride, and the fertilizer application rate was N 250 kg/hm^2^, P_2_O_5_ 325 kg/hm^2^, and K_2_O 150 kg/hm^2^.

### Soil sample collection

In late September 2021, the potato was in harvest, and the soil moisture content was between 19 and 24%. The rhizosphere soil of potato during the harvest period was collected by shaking soil method. After pulling out the tuber, the soil was shaken and dropped into the aluminum box. Five rhizosphere soil samples were collected from each test plot, and then mixed to form one soil sample and put into the aluminum box. Two aluminum box rhizosphere soils were collected from each test plot—one for agglomeration analysis and the other for soil properties. A total of 24 aluminum box soil samples was collected. The aluminum box soil for measuring soil properties was divided into two parts, one for chemical properties and the other for microbial analysis in the −80 °C refrigerator.

### Determination of soil microstructure

Remove water from the soil, cut off the dry soil sample, remove the extra grain and choose a relatively smooth part as the test plane. Apply electrically conductive adhesive to the work table, then apply the ion spray on the surface of the soil sample and place it in the sample chamber for analysis. The acceleration voltage is 10kV^31^. The magnification is 200 times.

### Soil aggregate measurement

Dry soil in an aluminum box indoors for later use. The water-stable aggregates were determined by the wet sieve method. The mechanical stability of aggregates was determined by the dry sieve method^32^.

### Determination of physical and chemical properties of soils

The soil organic matter (SOM), total nitrogen (TN), soil available phosphorus (SAP), soil available potassium (SAK), and soil texture were determined by potassium dichromate oxidation and external heating method, Kjeldahl nitrogen determination method, sodium bicarbonate extraction and molybdenum-antimony anti-spectrophotometry, sodium nitrate extraction and sodium tetraphenoboron turbidimetric method, and Malvern laser particle size analyze^33,34^.

### High throughput sequencing of 16S rRNA gene amplicon in soil samples

PCR amplification of V3-V4 variable regions was performed by primers 338F (5’-ACTCCTACGGGAGGCAGCAG-3') and 806R (5’-GGACTACHVGGGTWTCTAAT-3’). The PCR products were purified by 2% agarose gel, eluted by Tris–HCl, and detected by 2% agarose electrophoresis. Quanti FluorTM-ST (Promega, USA) was used for quantitative detection. Illumina's Miseq PE300 platform was used for sequencing^35^.

### Data processing

Excel 2019 was used to sort out the data and analyze the basic characteristics. The SPSS software (version v.19.0) was used to conduct statistical test on the test data (https://www.ibm.com/cn-zh/products/spss-statistics). Pearson correlation analysis was also conducted with SPSS 19.0 software. The composition of the bacteria community is based on the data table in tax_summary_a folder, which is drawn using R language tools. The diversity index was analyzed using Mothur (version v.1.30.2).

### Ethical approval

All procedures with plants were conducted in accordance with the guidelines and regulations.

## Data Availability

The datasets generated and/or analysed during the current study are available in the [INSDC] repository, and the sequencing data is available at **NCBI (SRA):** [SRR22797124, SRR22797123, SRR22797122, SRR22797121, SRR22797120, SRR22797119, SRR22797118, SRR22797117, SRR22797116, SRR22797115, SRR22797114, SRR22797113, SRR22797112, SRR22797111, SRR22797110, SRR22797109, SRR22797108, SRR22797107, SRR22797106, SRR22797105, SRR22797104, SRR22797103, SRR22797102, SRR22797101], **BioProject:** [PRJNA913429], **BioSample:** [SAMN32298774, SAMN32298775, SAMN32298776, SAMN32298777, SAMN32298778, SAMN32298779, SAMN32298780, SAMN32298781, SAMN32298782, SAMN32298783, SAMN32298784, SAMN32298785, SAMN32298786, SAMN32298787, SAMN32298788, SAMN32298789, SAMN32298790, SAMN32298791, SAMN32298792, SAMN32298793, SAMN32298794, SAMN32298795, SAMN32298796, SAMN32298797].
